# Dr. Exmon W. Earle

**Published:** 1914-10

**Authors:** 


Dr. Exmon W. Earle, Pulte Medical College, 1877, of Rochester, died at the Hahnemann Hospital, June 4, aged about 60.

